# Conveying Discovery to a Broad Audience

**DOI:** 10.1371/journal.ppat.1005425

**Published:** 2016-05-19

**Authors:** Laura J. Knoll

**Affiliations:** Department of Medical Microbiology and Immunology, University of Wisconsin–Madison, Madison, Wisconsin, United States of America; University of Notre Dame, UNITED STATES

Outcomes-based science promises results, like a cure for cancer or new antibiotics to treat infections. Such advances are important and exciting, but, unfortunately, they cannot be delivered in a short time frame. These miracle innovations are not certainties like the next software update, so our current emphasis on outcomes-based science is bound to disappoint the public. Not delivering on our promises is just one of the many reasons why the public does not trust scientists. Survey after survey, especially in America, shows that the general public has little trust in scientists. Are we all the evil mad scientists portrayed in the movies? When did “trust in scientists” become a political issue? How do we break down the walls and earn that trust back? How can the public trust scientists when they cannot understand us?

I have numerous examples of misunderstandings with my own family. I grew up on a farm in Minnesota. My dad attended school through the 8th grade, my mom stopped attending school at 11th grade, and none of my older siblings went to college. But, I got to play with the chemistry set my dad had bought for my brother, and I liked chemistry. Becoming a chemist was acceptable to my dad because I could get a respectable job at a company like 3M. Much to his dismay, I decided to leave chemistry to become a biochemist, then a parasitologist, and then an “ivory tower academic.” To my dad, these were incomprehensible moves—unacceptable choices I had made. He didn’t disown me, but he no longer bragged to his friends about me and a gap had formed between us. Why? Had I become an evil mad scientist? Could he no longer trust me? All I know is that he would ask me, “How are your little enzymes?” in response to which I would attempt to explain my current experiments. After a couple of minutes he would cut me off and say, “Well, next time you need help, make sure you come to me,” which was his way of telling me he was lost. He tried to understand, but I failed to explain. I got him lost in the trees instead of explaining why I loved the forest. I failed to convey my excitement for discovery.

What led me down each new trail in my career path was discovery: figuring out which chemicals I could mix together without making a mess in my brother’s room; learning about the complexity of biochemical pathways and their regulation; hearing about the intricacy of parasitic life cycles. How could parasites have evolved such convoluted life cycles and still survive after millions of years? Parasites are eukaryotic, like people, but they branch early off the evolutionary tree, so they have these mind-blowing processes, like editing their messenger RNA, having both mitochondria and remnant chloroplasts in the same cell, and being able to survive in the soil for years, or being able to survive in the human host without causing much damage. Determining how parasites evolved to exploit their unique environments requires that at least some hypothesis-generating experiments be performed.

The scientific method is (1) ask a question, (2) do background research, (3) construct a hypothesis, (4) test with an experiment, (5) analyze results, (6) draw conclusions, and (7) report results. I want to emphasize, “do background research.” Sometimes that background research is diving into the literature because, as the saying goes, “an hour in the library can save you a week in the lab.” But what if there is no literature pertaining to your scientific question? What if you work on an anciently diverging parasite for which 50%–60% of proteins are annotated as "hypothetical unknown?” Sometimes that background research has to be at the bench doing well-controlled but exploratory experiments that will allow you to construct a hypothesis. This hypothesis-generating science is what is missing and underappreciated with our current emphasis on translational or outcomes-based science. Some questions require background research at the bench without the constraints of a hypothesis to even imagine the scientific truth. Scientists must have the support to perform at least some discovery-based experiments, or we as discoverers will fail to make the next big breakthroughs. Scientists simply cannot guess the creative power of evolution.

My dad passed away before I got the chance to fill in the gap between us, but I know now that the key to the public understanding science is not in the details of experiments, but in the appreciation of wonder and discovery, even when its application to human health is not immediately obvious. My lab uses several different hypothesis-generating techniques to uncover how parasites establish and maintain chronic infection in their mammalian host and the consequences of that infection, good and bad, on the host. My lab has discovered parasite mechanisms that we would not have found unless we were performing hypothesis-generating studies. These mechanisms, and the excitement of their discovery, I have found quite easy to explain to the public.

We all have opportunities for public educational moments, big and small. Let’s not miss those educational moments that convey our enthusiasm for science and the time and energy it takes for the scientific method to come full circle on difficult problems. The necessity of discovery experiments and our passion for science is what needs to be translated to the public in order to bridge the gap and earn back their trust.

**Image 1:**
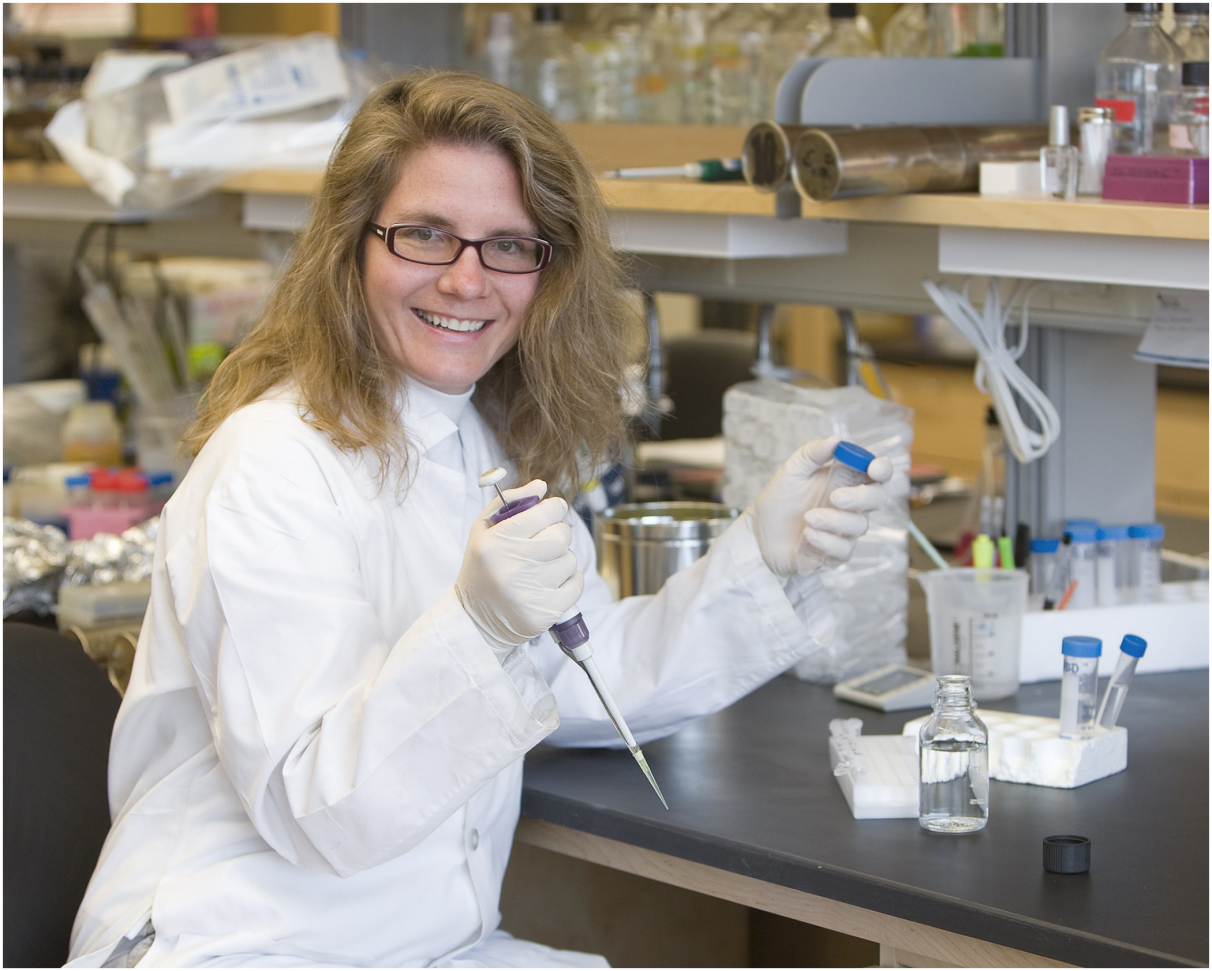
Laura J. Knoll.

